# ACLY facilitates colon cancer cell metastasis by CTNNB1

**DOI:** 10.1186/s13046-019-1391-9

**Published:** 2019-09-12

**Authors:** Jun Wen, Xuejie Min, Mengqin Shen, Qian Hua, Yuan Han, Li Zhao, Liu Liu, Gang Huang, Jianjun Liu, Xiaoping Zhao

**Affiliations:** 1 Department of Nuclear Medicine, Ren Ji Hospital, School of Medicine, Shanghai Jiao Tong University, Shanghai, China; 2 The First Affiliated Hospital of Nanyang Medical College, Nanyang, Henan Province China; 3 Department of Nuclear Medicine, Shanghai Chest Hospital, Shanghai Jiao Tong University, Shanghai, China; 4 Shanghai University of Medicine & Health Sciences, Shanghai, China

**Keywords:** ACLY, CTNNB1, Colon cancer, Metastasis, EMT (epithelial-mesenchymal transition)

## Abstract

**Background:**

Colon cancer is the second leading cancer worldwide. Recurrent disease and chemotherapeutic drug resistance are very common in the advanced stage of colon cancer. ATP-citrate lyase (ACLY), the first-step rate-controlling enzyme in lipid synthesis, is elevated in colon cancer. However, it remains unclear about the exact role of ACLY in the development of colon cancer metastasis.

**Methods:**

To evaluate the role of ACLY in colon cancer metastasis, we performed cell migration and invasion assays in two ACLY-deficient colon cancer cell lines. Colon cancer mouse model is used to examine ACLY’s effects on colon metastasis potentials in vivo. We analyzed the correlation between ACLY and CTNNB1 protein in 78 colon cancer patients by Pearson correlation. To finally explore the relationship of ACLY and CTNNB1, we used western blots, migration and invasion assays to confirm that ACLY may regulate metastasis by CTNNB1.

**Results:**

Our data showed that the abilities of cell migration and invasion were attenuated in ACLY-deficient HCT116 and RKO cell lines. Furthermore, we describe the mechanism of ACLY in promoting colon cancer metastasis in vitro and in vivo. ACLY could stabilize CTNNB1 (beta-catenin 1) protein by interacting, and the complex might promote CTNNB1 translocation through cytoplasm to nucleus, subsequently promote the CTNNB1 transcriptional activity and migration and invasion abilities of colon cancer cells. Immunohistochemical analysis of 78 colon cancer patients showed that the high expression levels of ACLY and CTNNB1 protein was positively correlated with metastasis of colon cancer.

**Conclusions:**

These results shed new light on the molecular mechanism underlying colon cancer metastasis, which might help in improving therapeutic efficacy.

**Electronic supplementary material:**

The online version of this article (10.1186/s13046-019-1391-9) contains supplementary material, which is available to authorized users.

## Background

Cancer statistics published by the American Cancer Society show that the incidence of human colon cancer is 10.2%, and its mortality rate has reached 9.2%, rising from the fourth place to the second [1, 2]. Generally, tumor resection is implemented for earlier stages of colon cancer. Combination of chemotherapeutic drugs is usually used in colon cancer patients at various stages, especially at the advanced stage. The chemotherapeutic drugs include cytotoxic drugs (5-fluorouracil, oxaliplatin, irinotecan and capecitabine) and biological agents (bevacizumab, panitumumab and cetuximab) [3]. Combination chemotherapy is initially effective in the majority of patients. Nevertheless, about 50% colon cancer patients develop recurrent disease due to drug resistance, and the 5 year survival rate decreases by more than 10% in advanced stage patients [4, 5]. Approximately 25% of colon cancer patients present with hepatic metastases on initial diagnosis, and about 50% will develop hepatic metastases within 3 years of the primary surgery [6]. Therefore, investigation of the molecular metastasis mechanism is crucial to address the unmet clinical need.

Tumor metastasis is a complex process. During metastasis, epithelial-derived tumor cells firstly acquire migration and invasion ability to diffuse from the primary tumor and enter into the blood circulation. Some surviving circulating tumor cells deform through the blood vessels and form distant metastasis. Tumor cells in colonization adjust themselves to adapt to the new microenvironment and switch from migration mode to proliferation mode in order to form metastases [7]. EMT plays an important role in the initial stage of metastasis. Although the process of metastasis is regulated by many respects. Metabolism reprogramming provides sufficient energy, amino acids, ribose, phospholipids and other macromolecules to meet demands for rapid proliferation [8], and it might play a critical role during the process of tumor metastasis.

Previously, researchers mainly focus on glucose and glutamine metabolism in the field of tumor metabolism and metastasis. There are few studies on lipid metabolism in tumor metastasis. Altered lipid metabolism in cancer cells has gradually attracted increasing attention in the past few years [9–11]. In contrast to normal cells mainly taking up fatty acids from the environment, tumor cells depend heavily on de novo lipogenesis [12]. ATP-citrate lyase (ACLY), an enzyme that generates acetyl-CoA from citrate, is the first-step rate-controlling enzyme responsible for the lipid synthesis [13]. And acetyl-CoA is carboxylated into malonyl-CoA, which signifies the first step of synthesizing fatty acids. Acetyl-CoA is also essential in the mevalonate pathway, in which two-molecular acetyl-CoA condenses into acetoacetyl-CoA, subquently synthesizing cholesterol [14]. In rapidly proliferating cancer cells, lipid synthesis and its intermediates are essential for cell membrane formation [15], corresponding signaling networks [16, 17], and tumor malignant progression [18]. However, there is little evidence of ACLY involvement in colon cancer metastasis.

CTNNB1 (which encodes beta-catenin 1 protein), a key Wnt signaling regulator, interacts with E-cadherin and actin cytoskeleton to mediate cell-cell adhesion [19]. CTNNB1 binds with APC, Axin, CK1 and glycogen synthetase kinase-3β (GSK3β) in the absence of Wnt ligands. CK1α and GSK3β phosphorylate CTNNB1 in turn, resultin g in ubiquitination and proteosomal degradation of CTNNB1 [20]. In the presence of Wnt ligands, CK1γand GSK3β mediate phosphorylation of LRP5/6 and Dsh, which jointly recruit Axin to the complex at the cell membrane. Due to the limited level of intracellular Axin [21], it is unable to form a degradation complex with CTNNB1. Cytoplasmic unphosphorylated CTNNB1 enters cellular nucleus, and functions as a transcriptional activator when complexes with members of the T-cell factor (TCF)/lymphocyte enhancer factor (LEF) family (LEF/TCF) of proteins [22]. In the nucleus, CTNNB1 forms a complex with LEF1, inhibits the transcriptional activity of E-cadherin, and induces epithelial-mesenchymal transition [23]. There are many studies on CTNNB1 in colon cancer [24, 25]. However, there is no published research on the relationship between ACLY and CTNNB1 in colon cancer.

In this study, we found ACLY promoted the ability of migration and invasion of colon cancer in vitro and in vivo. Furthermore, we identified that ACLY stabilized CTNNB1 protein by interacting, and the complex might promote CTNNB1 translocation through cytoplasm to nucleus, which promoted the CTNNB1 transcriptional activity and migration and invasion abilities of colon cancer cells. Our data shed new light on the molecular mechanism underlying colon cancer metastasis, which might help in improving therapeutic efficacy.

## Materials and methods

### Cell cultures

All cell lines were purchased from Cell Bank of the Chinese Academy of Science (Shanghai, China). SW480, DLD1, RKO, LOVO and HEK293T were cultured in Dulbecco’s modified Eagle’s medium (DMEM) (Gibco, USA) supplemented with 10% fetal bovine serum (FBS; Gibco, USA), 100 units/ml penicillin and 100 μg/ml streptomycin (Gibco, USA). HCT116 and its derivatives and HT29 were maintained in McCoy’s 5A Medium (Gibco, USA) supplemented with 10% FBS, 100 U/ml penicillin and 100 μg/ml streptomycin. Cells were cultured at 37 °C in a humidified incubator containing 5% CO_2_ and spread to multi-well plates at the peak of proliferation.

### Transfection of siRNA

SiRNA oligonucleotides and a negative control were designed and synthesized by GenePharma (China). Transfection was performed using lipofectamine 2000 (Invitrogen, USA) according to the protocol. The sequences of siRNA involved in this study are as follows: negative control (sense 5′-UUC UCC GAA CGU GUC ACG UTT-3′, antisense 5′-ACG UGA CAC GUU CGG AGA ATT-3′), ACLY (sense 5′-CGU GAG AGC AAU UCG AGA UUA-3′, antisense 5′-UAA UCU CGA AUU GCU CUC ACG -3′), CTNNB1–1 (sense 5′-GGG AGT GGT TTA GGC TAT TTG-3′, antisense 5′-CAA ATA GCC TAA ACC ACT CCC-3′), CTNNB1–2(sense 5′-UUG UUA UCA GAG GAC UAA AUA-3′, antisense 5′-UAU UUA GUC CUC UGA UAA CAA-3′).

### Construction of the ACLY-knockout and shACLY cell lines

To construct the HCT116 ACLY-knockout (KO) cell line, guide RNA sequence was designed by Clustered Regularly Interspaced Short Palindromic Repeats Cas9 system technology (CRISPR Cas9) designer at www.crispr.mit.edu. The single guide RNA sequence (sgRNA) was 5′-GAGCATACTTGAACCGATTC-3′, cloned into lentiCRISPRv2 plasmid, which was transfected with viral packaging plasmids (pspa and pmd2g) into HEK293T cells to generate lentivirus. Forty-eight hours after infection with viral supernatant, selection was performed with puromycin (1 μg/ml) for 3 days to obtain single-cell KO clones, which were further expanded. Loss of ACLY was proved by DNA sequence (data was shown in Additional file 2) and western blot. The negative control cells were transfected with the lentiCRISPRv2 plasmid not containing the guide sequence. The process of selecting single-cell normalization control clones (Ctrl) was similar to the KO cell line. The siRNA-ACLY oligonucleotide (5′-CGT GAG AGC AAT TCG AGA TTA-3′) was cloned into pLV-U6 plasmid, which was co-transfected with packaging plasmids (pspa and pmd2g) into HEK293T cells. 72 h after transfection, the medium with viral supernatant was harvested and filtered through a 0.45 μm strainer. RKO cells were infected by viral supernatant. The negative control group was infected with siRNA-NC (5′-UUC UCC GAA CGU GUC ACG UTT-3′) viral supernatant. The process of selection was the same as the HCT116 KO cell.

### Quantitative real-time PCR (qPCR)

Total RNA was isolated using the TRIzol kit (Omega, USA), and transcribed to cDNA immediately using the Prime-Script RT kit (Takara, China). Quantitative real-time PCR was carried out in 96-well plates using the StepOne Plus Real-Time PCR System (Applied Biosystems, USA). After immediate reverse transcription, cDNA was amplified using SYBR Green PCR Master Mix (Takara, Japan). Each experiment was performed in triplicate. Cyclophilin B (CB) was used as the control. The primer sequences are shown in Additional file 1: Table S1.

### Western blot

To obtain total protein lysates, fresh tissue was ground to powder by adding lipid nitrogen. Grinding tissue or cell precipitates were lysed using cell lysates containing mixed proteinase inhibitors. The lysate was incubated on ice for 30 min followed by centrifugation at 4 °C 15000 g for 30 min. The protein concentration of each sample was assayed using the bicinchoninic acid (BCA) assay. The cytoplasmic and nuclear extracts were separated by extraction regents (Invitrogen, USA). Samples were loaded on 7.5% or 10% SDS-PAGE. After electrophoresis, the proteins were transferred from gels to PVDF membranes. The membranes were blocked in 5% low-fat dried milk in TBST for an hour at room temperature and then incubated with the primary antibodies overnight at 4 °C. The primary antibodies included anti-ACLY (Proteintech, 15,421), anti-CTNNB1 (Cell Signaling Technology, 8480), anti-E-cadherin (Cell Signaling Technology, 3195), anti-N-cadherin (Cell Signaling Technology, 13,116), anti-Vimentin (Cell Signaling Technology, 5741), anti-ZO-1 (Cell Signaling Technology, 8193), anti-Snail (Cell Signaling Technology, 3879), anti-β-actin (Cell Signaling Technology, 12,262), anti-Flag M2 (Sigma, A2220), anti-Lamin B1 (Proteintech, 66,095). The immunoreactive bands were visualized by the ECL Plus system (Tanon, China).

### Immunoprecipitation (IP)

Anti-Flag M2 affinity gel was incubated with cell extracts overexpressing Flag-tagged ACLY proteins at 4 °C for 3 h. For endogenous co-immunoprecipitation assay, 15 μl protein A/G agarose was incubated with antibody against ACLY and HEK293T cell extracts in turn. The protein A/G agarose should be washed at least three times with IP buffer after the first incubation, in order to completely wash off the remaining antibodies. The samples were subjected to western blot.

### Transwell assay

Cells cultured in 200 μl medium without FBS were seeded into the upper chamber of matrigel-coated (8 μm pore size chamber inserts; Corning, USA) or uncoated membrane filters. The mambrane was coated matrigel in transwell invasion assay. The lower chamber was filled with 500 μl medium with 10% FBS. Then, cells were maintained in the incubator at 37 °C 5% CO_2_ for 48 h. Cells migrated through the membrane were fixed with 4% formalin for 30 min, stained with 0.1% crystal violet for 2 min and washed with PBS to weaken the background. Images of five random fields were captured using an upright microscope (magnification, × 100).

### Wound healing assay

Cells at the peak of proliferation were seeded in six-well plates to 95% confluence. A linear wound was scratched with a 200 μl sterile pipette tip across the monolayers when cells were seeded for 24 h. After washing with phosphate-buffered saline (PBS) to remove the cell debris, the adherent cells were incubated in medium with 1% FBS at 37 °C 5% CO_2_. The wounded monolayers were photographed at 0, 24 and 48 h after scratching.

### Triglyceride and cholesterol assay

Cells were washed twice with PBS and lysed. Total protein concentration was detected using the bicinchoninic acid assay kit (Abcam). Intracellular triglyceride and cholesterol were measured by using the triglyceride assay kit (Biovision, USA) and cholesterol assay kit (Biovision, USA) according to the protocol.

### Oil red O staining

Cells were seeded in six-well plates, and then fixed by 4% paraformaldehyde for 30 min. Then, cells were permeabilized in 60% isopropanol for 10 s, stained with Oil Red O working solution at room temperature for 5 min, and washed cells with 60% isopropanol for 10 s again. The cells were rinsed with distilled water and photographed using a Zeiss Axioskop microscope.

### Immunofluorescence staining

The Ctrl and KO HCT116 cells were seeded in six-well plates. After 24 h, cells were fixed in 4% paraformaldehyde at room temperature for 30 min. Cells were incubated with anti-E-cadherin antibody (1:100; Cell signaling technology) and DAPI-Fluoromount-G (Southern Biotech). The protocol was described in detail in our previous study [26].

### Immunohistochemistry

Colon tumor specimens were resected from patients who signed informed consent at Renji Hospital, Shanghai Jiao Tong University School of Medicine. Only 63 of 78 patients had follow-up records. The follow-up time ranged from 0.4 to 78 months, with a median time of 51.0 months. Tumor tissues were fixed and embedded in paraffin. IHC analysis was performed as described previously [26]. The primary antibodies used were anti-ACLY (1:100, Proteintech) and anti-CTNNB1 (1:200, CST). The slides were scored by two independent investigators without prior knowledge about the patients. Signals in tumor cells were quantified using a scoring system ranging from 0 to 9. The signal intensity was scored as 0 (no signal), 1 (weak signal), 2 (moderate signal) or 3 (strong signal), and the percentage positive staining was scored as 0 (< 10%), 1 (< 25%), 2 (< 50%) or 3 (≥50%). High expression level was defined as IHC score was more than 5.

### Luciferase reporter assay

To confirm that CTNNB1 was a target gene of ACLY in colon cancer cells, we used a pair of luciferase reporter plasmids, TOP-flash (with 3 repeats of the TCF-binding site) and FOP-flash (with 3 repeats of the TCF-binding site), which were widely used to evaluate CTNNB1 transcriptional activity. Firefly and renilla luciferase plasmid were transfected into cells at a ratio of 30:1 using lipofectamine 2000. After 24 h, firefly and renilla luciferase activities were analyzed using the Dual-Luciferase Reporter Assay System (Promega).

### Xenograft metastasis assays

All experiments were performed in vivo abiding by the guidelines of the Animal Ethics Committee of Renji Hospital, School of Medicine, Shanghai Jiao Tong University. We constructed the experimental metastatic model by tail vein injecting 1.5 × 10^6^ HCT116 cells in BALB/C-nu/nu male mice (5 weeks; Shanghai Laboratory Animal Center, China). Nude mice were kept in a sterile environment. After eight weeks, each of them was scanned by a micro PET-CT (Pingseng Healthcare Co, China) with being injected 200 μCi ^18^F-FDG. Maximum standardized uptake (SUVmax) of regions of interest (ROI) were evaluated after manual definition. Then they were euthanized by cervical dislocation. Lung and liver tissues were fixed with 4% paraformaldehyde. Tissues were embedded in paraffin and stained with hematoxylin and eosin (HE), Ki67, ACLY and CTNNB1 antibody.

### Statistical analysis

Data were analyzed using GraphPad Prism 7 or SPSS 23.0. Statistical differences between groups were evaluated by the two-tailed t-test. Pearson correlation analyses were performed on the correlation between ACLY and CTNNB1. Kaplan–Meier analysis was used for survival analysis. *P < 0.05 from a two-tailed test was considered as significant. **P < 0.01, ***P < 0.001.

## Results

### ACLY promotes metastasis in the colon cancer cells in vitro

**Fig. 1:**
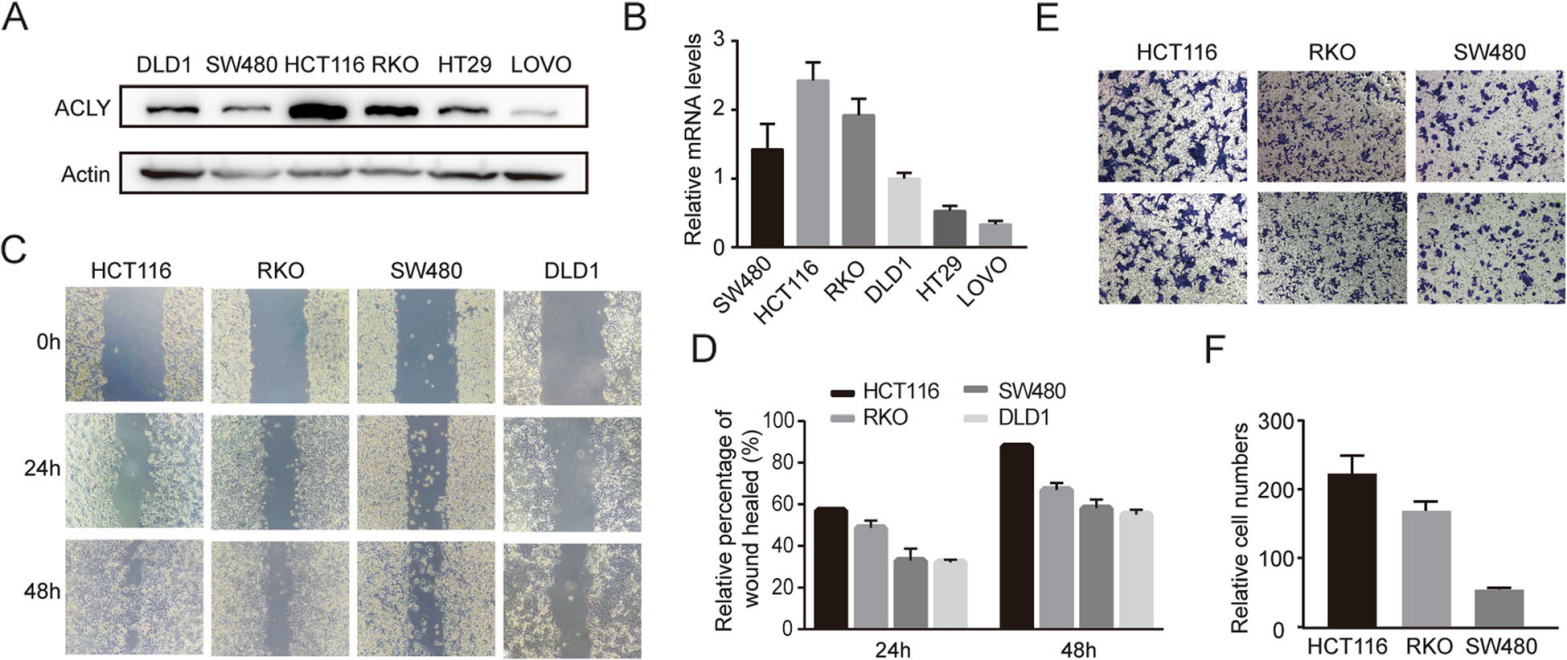
High expression levels of ATP-citrate lyase (ACLY) may promote colon cancer metastasis in vitro. **a**, **b** Western blot (**a**) and qPCR (**b**) analysis of ACLY in six colon cancer cell lines. Actin was the loading control. **c** Wound healing assays to access the migration ability of HCT116, RKO, SW480 and DLD1. **d** The quantitative analysis of wound healing rates. **e** Transwell invasion assays to evaluate the invasion ability of HCT116, RKO and SW480. **f** The quantitative analysis of cells across the transwell membrane

Firstly, we analyzed the endogenous protein and mRNA levels of ACLY in six human colon cancer cell lines (Fig. 1a and b). HCT116, RKO and SW480, expressing higher levels of ACLY, were chosen to investigate the significance of ACLY in the process of colon cancer metastasis by the wound healing assay and Transwell invasion assay. As shown in Fig. 1c and d, the cell migration ability of HCT116, RKO, SW480 and DLD1 weakened in sequence, compared with the wound healing rates after scratching 24 or 48 h. Transwell invasion assays showed that the invasion ability of HCT116 was stronger than RKO and SW480 (Fig. 1e and f). These results indicate that the expression levels of ACLY might be positively correlated with the invasive and metastatic abilities.

### ACLY deficiency inhibits the cell lipid formation and metastasis

**Fig. 2:**
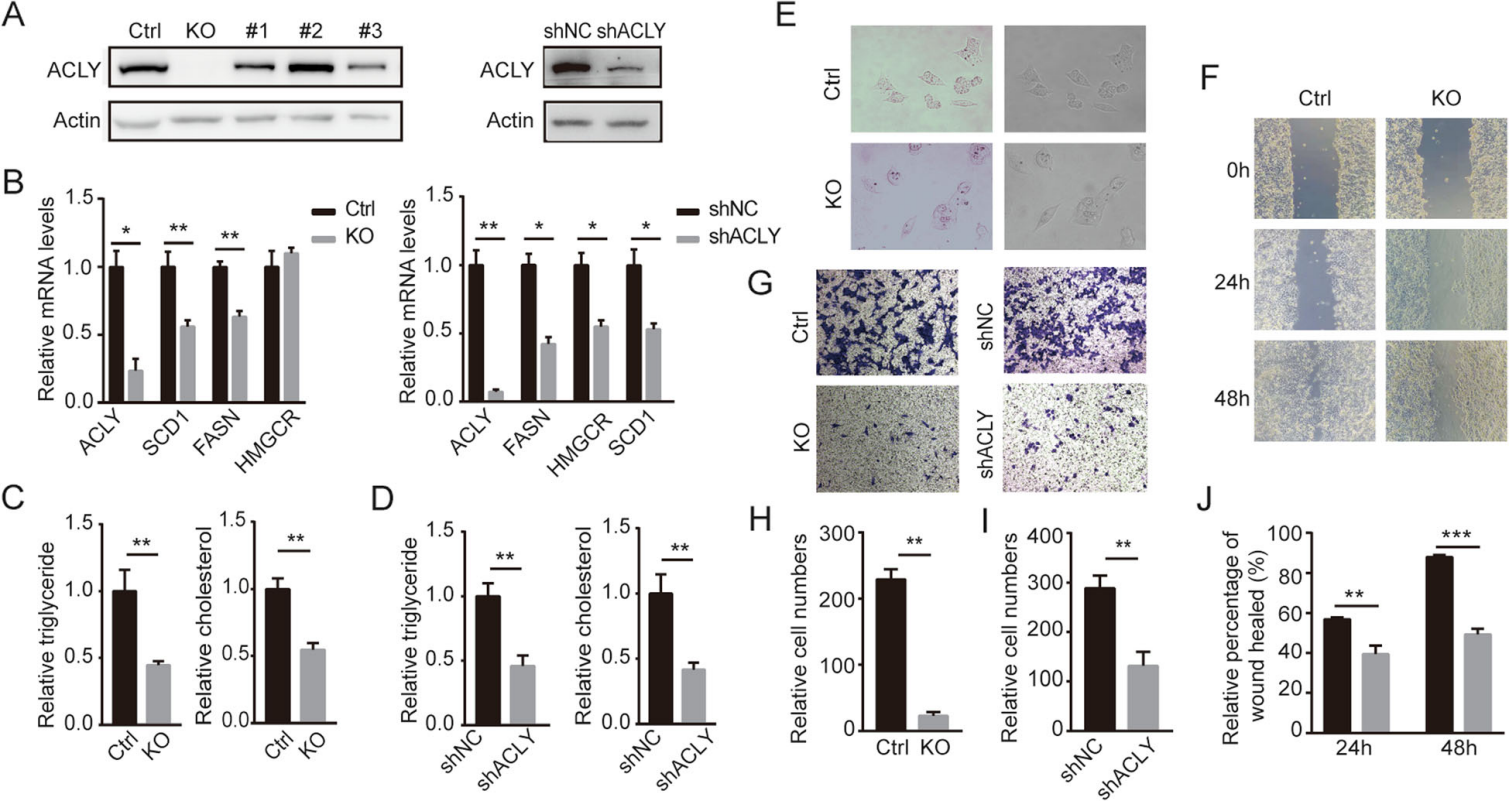
ACLY deficiency weakened lipid formation and metastasis of colorectal cancer cell. **a** Western blot analysis to access the efficiency of ACLY knockout in HCT116 cells and ACLY knockdown in RKO cells. Actin was the loading control. Ctrl: the normal control, KO: ACLY-knockout. #1-#3: the signal-cell clones that did not knock out ACLY in HCT116 cells. shNC: RKO signal-cell clones infected with negative control shRNA virus. shACLY: RKO signal-cell clones infected with shRNA-ACLY virus. **b** Real-time PCR analysis to qualify the changes of mRNA expression of the major lipid synthase when ACLY deficiency in HCT116 and RKO cells. SCD1: stearoyl-CoA desaturase 1, FASN: fatty acid synthase, HMGCR: 3-hydroxy-3-methylglutaryl-CoA reductase. **c**, **d** The quantitative detection of triglyceride and cholesterol when ACLY deficiency in HCT116 and RKO cells. **e** HCT116 cells stably knocked out ACLY and the control cells were used for Oil Red O staining (magnification, 200×). **f**, **j** Wound healing assays to access the effects of ACLY deficiency on the migration ability of HCT116 cells. **g**, **h** Transwell invasion assays to evaluate the effects of ACLY deficiency on the invasion ability of HCT116 and RKO cells. The quantitative analysis of cells across the transwell membrane (**h** and **i**). The quantitative analysis of wound healing rates (**j**)

To further investigate the effects of ACLY deficiency on colon cancer metastasis, we applied the CRISPR Cas9 technology to knock out endogenous ACLY gene in HCT116 cells (KO), and we also obtained RKO cells that stably knocked down ACLY (shACLY) by shRNA-ACLY. The protein level of ACLY was verified by western blot (Fig. 2a). Quantitative real-time PCR (qPCR) confirmed that ACLY deficiency resulted in suppression of primary lipogenic enzymes, such as stearoyl-CoA desaturase 1 (SCD1), fatty acid synthase (FASN). The mRNA level of 3-hydroxy-3-methylglutaryl-CoA reductase (HMGCR) decreased in RKO shACLY cells, but had no significant changes in HCT116 KO cells (Fig. 2b). Compared with HCT116 normal control cells, the levels of triglycerides and cholesterol in ACLY knockout cells were notably reduced (Fig. 2c). Similar changes were observed in RKO cells (Fig. 2b and d). Lipid droplets, detected by Oil Red O staining, were less abundant in ACLY KO cells than in the control (Fig. 2e). As shown in Fig. 2f-j, loss of ACLY substantially reduced the cell motility and invasion ability compared to the control in wound healing assay and Transwell invasion assay. Taken together, colon cancer cells deficient ACLY have reduced lipid synthesis and weakened metastatic abilities.

### ACLY deficiency impaired metastasis of colon cancer cells in vivo

**Fig. 3:**
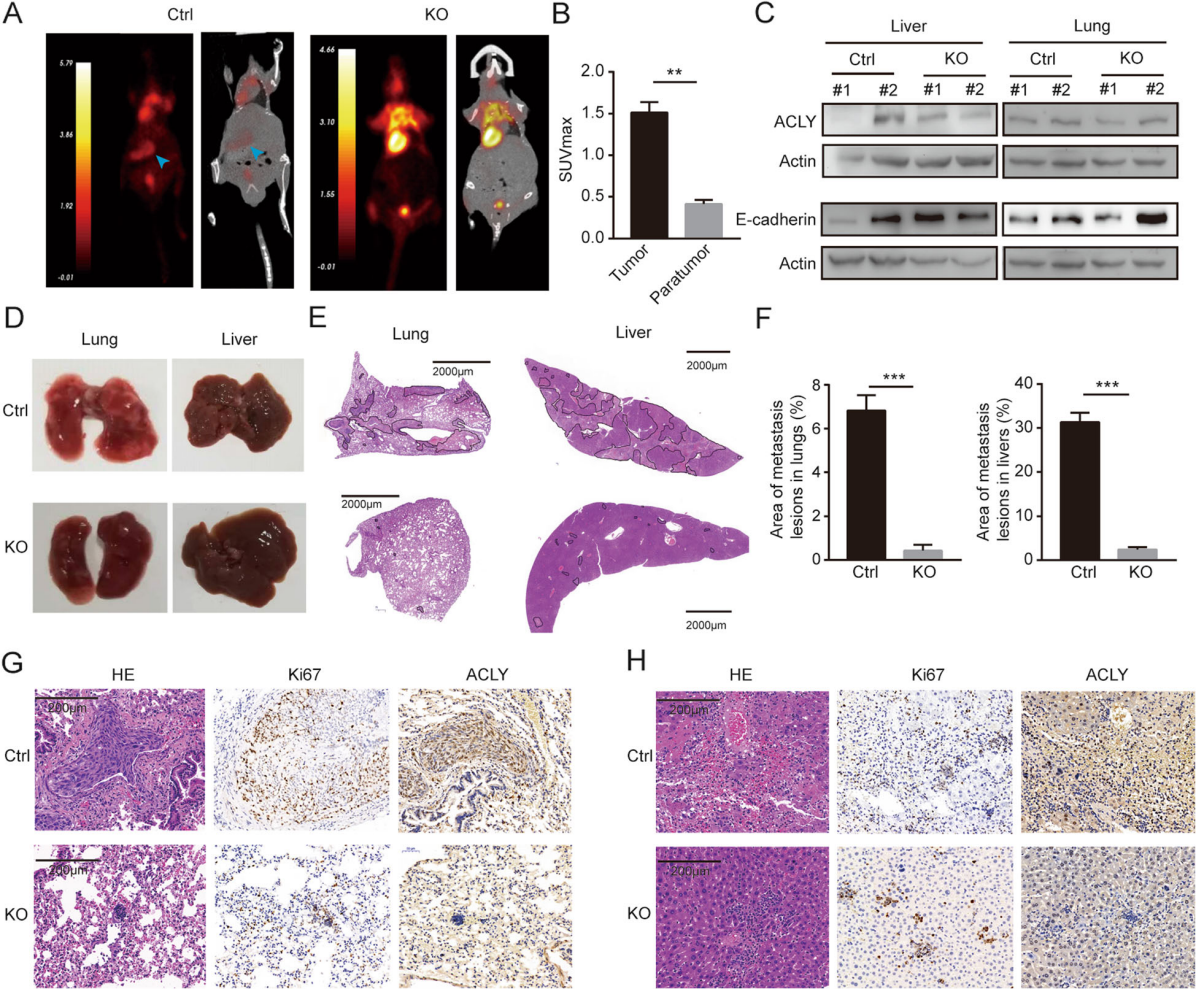
Loss of ACLY significantly weakened the invasive ability in vivo. **a** The PET or PET-CT images of nude mice metastasis model by tail vein injection of ^18^F-FDG. The arrow pointed to the high metabolite of ^18^F-FDG concentration. **b** Comparison of the maximum standard ^18^F-FDG uptake value (SUV max) between tumor tissues and surrounding normal tissues in the control group. **c** The protein levels of ACLY and E-cadherin in liver and lung tissues of the control and KO group detected by western blot. Actin was the loading control. **d**-**h** Pictures of representative lung or liver tissues (D) and tissue sections of intrahepatic and pulmonary metastases (**e**) were shown, the area quantification of intrahepatic and pulmonary metastatic foci was analyzed by Student’s t-test (**f**). Pictures of representative HE staining, Ki67 staining and ACLY staining of metastatic lesion and precancerous tissue in lungs (**g**) and livers (**h**). Scale bar: 2000 μm (**e**); 200 μm (**g** and **h**)

The nude mouse metastasis model was established to investigate the motility and invasion abilities of colon cancer cells deficient ACLY in vivo. After 8 weeks, the nude mice were performed micro PET-CT imaging by tail vein injection of ^18^F-FDG. As shown in Fig. 3a, high-metabolism lesions were found in livers of the control group, which showed the concentration of radionuclide ^18^F-FDG. And the maximum standard uptake value (SUV max) was significantly higher than that of surrounding normal tissues (Fig. 3b). Whereas, no obvious concentration of ^18^F-FDG was found in the KO group. Additionally, no obvious concentration was observed in the lungs, where metastasis lesions might be small or interfered by the high ^18^F-FDG uptake of the heart. Histology showed that the lungs of the control group were observed a large amount of white clump-like dense tissues, while no obvious clumps were observed in the KO group. And the surface of livers was not smooth, uneven and the elasticity was worse than the KO group (Fig. 3d). According to the hematoxylin-eosin staining (HE) immunohistochemical staining, the metastatic lesion area of livers and lungs was obviously smaller in the KO group than control (Fig. 3e and f). In the liver and lung metastatic lesions of the control group, the ACLY protein level was notably higher than the KO group. Moreover, strong Ki67 expression in the lung and liver of the control group suggested that ACLY promoted the growth of metastatic cells (Fig. 3g and h). Furthermore, we detected a decrease of ACLY protein level and an increase of E-cadherin protein level in the liver and lung tissues of the KO metastasis model, compared with the control group (Fig. 3c). These findings suggest that ACLY deficiency weakens the colon cancer cell metastasis ability in vivo.

### ACLY is involved in process of epithelial-mesenchymal transition

One of the behavioral characteristics of malignant tumors is the spread of tumor cells. These invasive cells undergo a transformation from epithelial cells to mesenchymal cells (epithelial-mesenchymal transition, EMT) [27], which plays an important role in the initial stage of metastasis. During EMT, tumor cells lose epithelial cell characteristics (motility limitation and cell-cell strong junctions), while acquiring mesenchymal characteristics associated with individual invasion (motility enhancement, cell-cell weak junctions and spindle-like morphology) [28]. However, there are few studies on the relationship between lipid metabolism enzymes and EMT. Here we tried to explore the effects of lipid synthesis key enzyme ACLY on metastasis, especially on EMT.

**Fig. 4:**
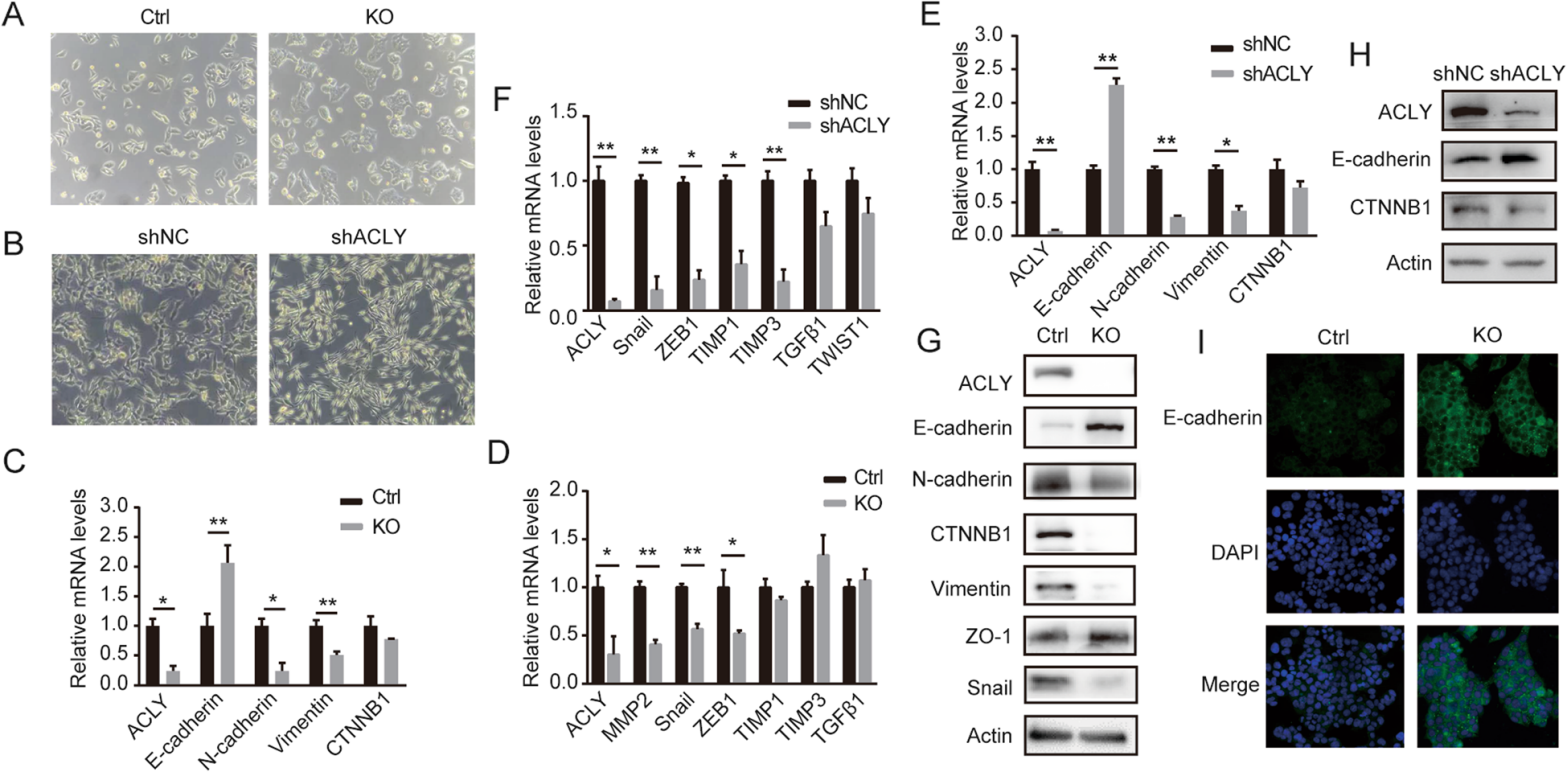
ACLY affects epithelial-mesenchymal transition (EMT) of CRC in vitro. **a**, **b** Image showed morphological changes of HCT116 and RKO cells stably knockdown ACLY vs control cells (magnification, 200×). **c**-**f** Quantitative real-time PCR (qPCR) analysis to qualify the endogenous mRNA levels of EMT-associated markers (**c**, **e**) and transcriptional factors (**d**, **f**) in HCT116 and RKO cells ACLY deficiency or the control. **g**, **h** Western blot to determine the protein levels of EMT markers and transcriptional factors in HCT116 and RKO cells ACLY deficiency or the control. Actin was the loading control. **i** Immunofluorescence assays showed the E-Cadherin protein level in HCT116 control cells was lower than KO cells (magnification, 200×)

At first, we observed that knocking out ACLY was associated with changes of the mesenchyme-like morphology of HCT116 cells to an epithelium-like morphology, such as a decrease in scattering and adoption of a spindle-shaped shape (Fig. 4a). This phenomenon suggested that ACLY may be associated with the epithelial-mesenchymal transition process. To further evaluate the role of ACLY in EMT, we detected the mRNA levels of some EMT marker genes. Comparing with HCT116 control cells, there was an increase of the E-Cadherin mRNA level, and significant decreases of mRNA levels of mesenchymal genes (N-cadherin and Vimentin) in HCT116 KO cells (Fig. 4c). We also noted EMT-associated transcriptional factors Snail, ZEB1 and MMP2 were attenuated in HCT116 cells stably knocked out ACLY (Fig. 4d). Similar phenomenon was observed in RKO cells (Fig. 4b, e and f). Consistently, knocking out ACLY resulted in increasing E-Cadherin protein level and decreasing N-cadherin, CTNNB1, Vimentin and snail protein levels (Fig. 4g). Immunofluorescent staining showed that the protein level of E-Cadherin increased in the KO cells (Fig. 4i). We also detected the increased E-Cadherin and decreased CTNNB1 protein levels in RKO cells (Fig. 4h). These observations suggest that ACLY promotes the process of metastasis in colon cancer cells, especially the process of epithelial-mesenchymal transition.

### The relationship of ACLY and CTNNB1 in colon cancer patients

**Fig. 5:**
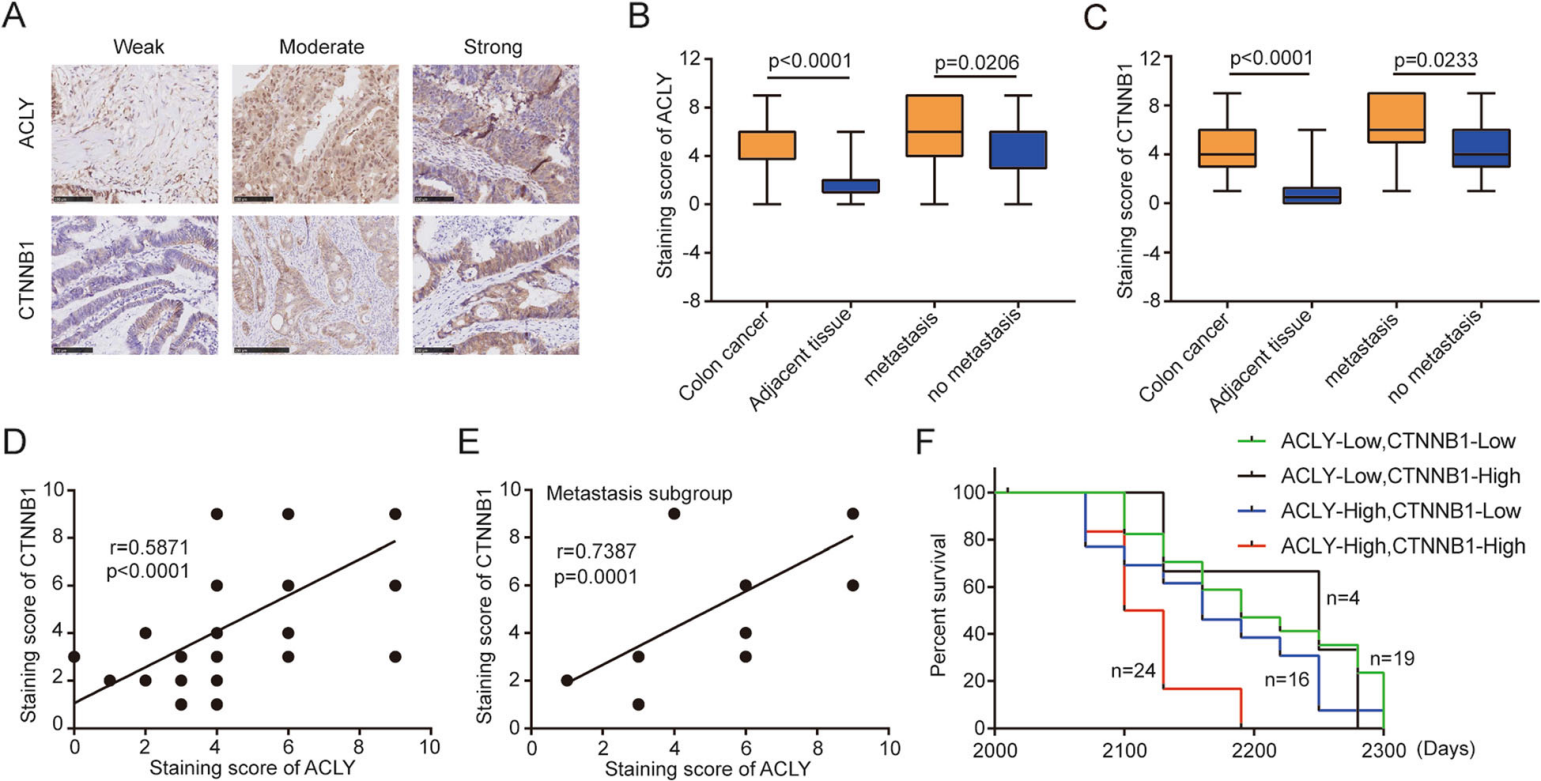
ACLY is positively correlated with CTNNB1, both correlated with tumor metastasis and patient survival. **a** Representative images of staining with ACLY and CTNNB1 antibodies in colon cancer tissue showed strong, moderate, and weak expression, respectively (scale bar: 100 μm). **b**, **c** Statistical analysis of ACLY staining (**b**) and CTNNB1 staining (**c**) between colon cancer tissues and adjacent normal tissues or metastasis and no-metastasis tissues. **d** Pearson correlative analysis of staining scores for ACLY and CTNNB1. **e** Pearson correlative analysis of staining scores for ACLY and CTNNB1 in the metastasis subgroup. **f** Kaplan-Meier overall survival analysis of colon cancer patients. ACLY^high^CTNNB1^high^ (red curve), ACLY^high^CTNNB1^low^ (Blue curve, *P* = 0.0005), ACLY^low^CTNNB1^high^ (Black curve, *P* = 0.0674) and ACLY^low^CTNNB1^low^ (Green curve, *P* < 0.0001) group was showed (*P* value is obtained by Kaplan–Meier analysis between ACLY^high^CTNNB1^high^ group and other groups)

Seventy-eight pairs of colon cancer tissues were examined by IHC (scale bar, 501 μm) (Fig. 5a). The ACLY staining score of colon cancer tissue was significantly higher than adjacent tissue (*P* < 0.0001). And the ACLY score of metastasis subgroup was also higher than the no metastasis subgroup (*P* = 0.0206) (Fig. 5b). A similar situation appeared in CTNNB1 (Fig. 5c). As shown in Table 1, there was no notable difference in ACLY or CTNNB1 expression with age and gender. The protein level of ACLY and CTNNB1 was also related to lymphnode metastasis. Interestingly, we found that ACLY was positively related to CTNNB1 in colon cancer tissues (*r* = 0.5871, *P* < 0.001) (Fig. 5d). In the metastasis subgroup, there existed a strong correlation between ACLY and CTNNB1 (*r* = 0.7387, *P* = 0.0001) (Fig. 5e). Compared with patients with low-level ACLY and CTNNB1, patients with high levels of ACLY and CTNNB1 had poorer survival than low levels of ACLY and CTNNB1 (Fig. 5f). Therefore, these results indicate that high levels of both ACLY and CTNNB1 play a critical role in the metastasis of colon cancer.

**Table 1:** Analysis of correlation between ACLY or CTNNB1 protein level and clinic parameters in 78 patients with colon cancer

Protein name		ACLY			CTNNB1		
Characteristics	All cases	Low	High	*P* value	Low	High	*P* value
Participants	78	32	46		44	34	
Age (years)				0.058			0.712
<60	27	15	12		16	11	
≥60	51	17	34		28	23	
Gender				0.414			0.751
Male	42	19	23		23	19	
Female	36	13	23		21	15	
AJCC clinical stage				0.081			0.040
1–2	50	25	25		33	17	
3–4	28	7	21		11	17	
Tumor size (mm^3^)				0.516			0.117
<30	38	17	21		18	20	
≥30	40	15	25		26	14	
Lymphnode metastasis				0.049			0.042
Negative	51	25	26		33	18	
Positive	27	7	20		11	16	

### ACLY promotes colon cancer metastasis via promoting CTNNB1 translocation to nucleus

**Fig. 6:**
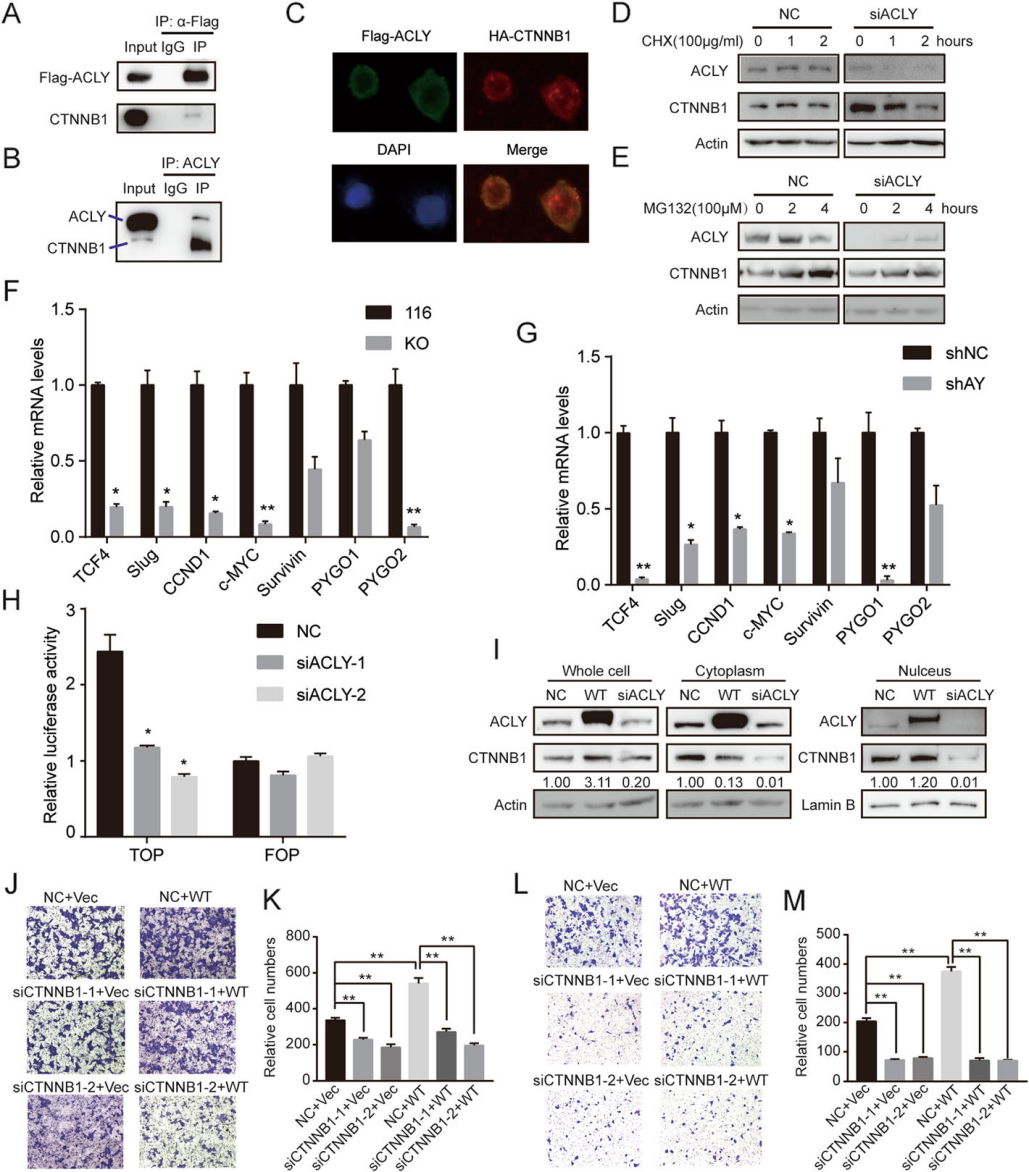
ACLY promoted colon cancer metastasis via promoting CTNNB1 translocation to nucleus. **a** HEK293T extracts transfected with Flag-ACLY for 48 h were immunoprecipitated with anti-Flag or mouse IgG and immunoblotted by anti-CTNNB1. **b** HEK293T extracts were immunoprecipitated with anti-ACLY or rabbit IgG and immunoblotted by anti-CTNNB1. **c** After 48 h of transfection, the colocation (yellow) of exogenous Flag-ACLY (green) and HA-CTNNB1 (red) in HEK293T cells were analyzed using a fluorescence microscope (magnification, 400×). Cell nucleus was stained by DAPI (blue). **d** HCT116 cells were transfected with siRNA-NC or siRNA-ACLY for 48 h, followed by 0, 1 and 2 h cycloheximide (CHX; 100 μg/ml) treatment or DMSO. Cell lysates were immunoblotted with anti-ACLY or anti-CTNNB1. Actin was the loading control. **e** HCT116 cells were transfected with siRNA-NC or siRNA-ACLY for 48 h. MG132 (100 mmol/l) was added for 2, 4 h or DMSO. Cell lysates were immunoblotted with anti-ACLY or anti-CTNNB1. Actin was the loading control. **f**, **g** mRNA levels of the indicated genes [TCF4, Slug, CCND1, c-MYC, Survivin, PYGO1, PYGO2] in ACLY stably silenced HCT116 cells and RKO cells were analyzed by QPCR. (H) Luciferase reporter assay using Top-flash and Fop-flash vectors was used to study CTNNB1 TCF promoter activity. 293 T cells were transfected with siACLY-1 or siACLY-2 (or siRNA-NC) for 48 h before luciferase reporter assay. **i** HCT116 cells transfected with siRNA-NC, siRNA-ACLY or Flag-ACLY for 48 h. Part of the cells was used to extract nuclear and cytosolic fractions. Cell lysates were immunoprecipitated with anti-ACLY or anti-CTNNB1. CTNNB1 band intensity was normalized to actin. Actin was the loading control. **j**-**m** HCT116 cells were cotransfected with Flag-ACLY or empty vector (as control) plus siRNA-NC or siRNA-CTNNB1 for 48 h. Efficiency of knockdown CTNNB1or overexpression of ACLY was assayed by western blot (Additional file 3: Figure S6B). Transwell migration assay (**j**, **k**) and invasion assay (**l**, **m**) were performed in HCT116 cells cotransfected with Flag-ACLY (or vector as control) and siCTNNB1 (or siNC as control). The quantitative analysis of cells across the transwell membrane to access the migration and invasion abilities (**k** and **m**)

CTNNB1 is a key regulator involved in the process of epithelial-mesenchymal transition. The finding that the ACLY and CTNNB1 synergistically promote colon cancer metastasis led us to further examine their relationship. Interestingly, we found that ACLY protein can interact with CTNNB1 protein. It was confirmed by co-immunoprecipitation assays at both endogenous and exogenous levels (Fig. 6a and b). And flag-tagged ACLY was colocalized with HA-tagged CTNNB1 (Fig. 6c). Furthermore, protein synthesis inhibitor cycloheximide (CHX) was used to observe the degradation of CTNNB1 (Fig. 6d). Results showed that ACLY knockdown (siACLY) caused faster degradation of CTNNB1 than the negative control group (NC). MG132 was added to inhibit the degradation of CTNNB1, which was more effective when ACLY was not knockdown (Fig. 6e).

To further explore whether ACLY regulates CTNNB1, we analyzed the transcript levels of CTNNB1 and its target genes in ACLY stably silenced HCT116 cells and RKO cells (Fig. 6f and g). The target genes of CTNNB1, including TCF4, Slug, CCND1, c-MYC, Survivin, PYGO1, PYGO2, were downregulated by ACLY silenced to different degrees. HCT116 and RKO cell lines showed a similar phenotype. Some CTNNB1 target genes were differently regulated by ACLY in different colon cancer cells. We then used TOP-flash and FOP-flash luciferase reporter plasmids, which were widely used to evaluate CTNNB1-dependent signaling activity in HEK293T and HCT116 cells. As shown in Fig. 6h and Additional file 3: Figure S6A, ACLY knockdown decreased the transcriptional activity of CTNNB1. These results suggest that ACLY could upregulate CTNNB1 transcriptional activity.

**Fig. 7:**
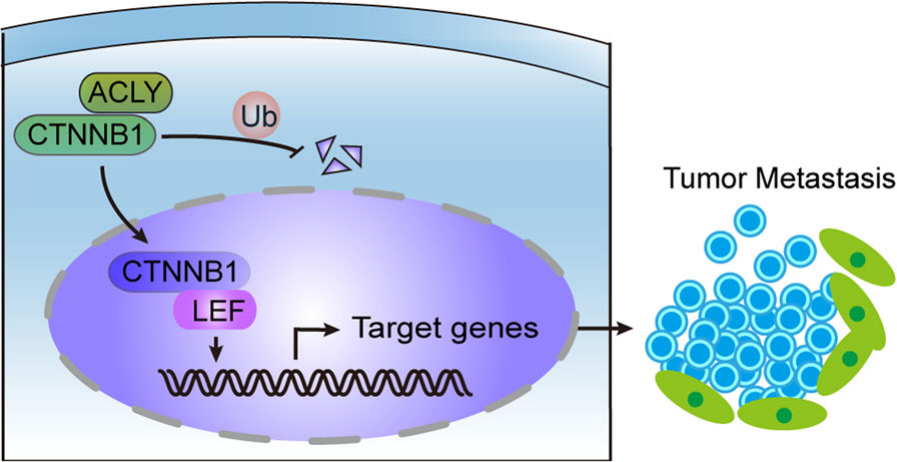
Schematic representation of ACLY and CTNNB1 implicated in colon cancer metastasis. ACLY interacted with CTNNB1 and might block the ubiquitination of CTNNB1, and subsequently promoted CTNNB1 translocation through cellular cytoplasm to nucleus. The binding of CTNNB1 to LEF in the nucleus further activated transcription factors such as Snail and repression of E-cadherin, promoting the metastasis of tumor cells. ACLY: ATP citrate lyase, Ub: ubiquitination, LEF: lymphoid enhancer-binding protein

To explore how ACLY regulated CTNNB1, quantification of CTNNB1 protein were analyzed by western blot in different intracellular locations of ACLY-overexpressed and ACLY-knockdown HCT116 cells (Fig. 6i). Results showed that ACLY overexpression increased the protein level of CTNNB1 in the whole cell, especially in nuclear fractions. Conversely, knocking out ACLY reduced the protein level of CTNNB1 in the whole cell, including in cytoplasm and nuclear fractions. Moreover, we found that knockdown of CTNNB1 weakened the effects of ACLY on colon cancer cell migration and invasion (Fig. 6j-m). According to these data, we formulated a hypothesis that ACLY could stabilize CTNNB1 protein by interacting with it, and the complex might promote CTNNB1 translocation through cytoplasm to nucleus, which promotes the CTNNB1 transcriptional activity and migration and invasion abilities of colon cancer cells. To make it visually accessible, we drew a schematic to describe the hypothesis (Fig. 7).

## Discussion

Rapidly proliferating tumor cells are in great demand for macromolecules, such as lipids. Since circulating lipids cannot meet the demand, cancer cells dramatically increase de novo lipogenesis [29]. The excessive accumulation of intracellular fatty acids not only contributes to the synthesis of biologic membrane in tumor cells and signaling transduction, but also promotes the process of metastasis [18]. ACLY, the first-step rate-controlling enzyme, plays a critical role in the lipid synthesis. High ACLY protein level was correlated with advanced stages and lymph node metastasis in gastric adenocarcinoma [30]. Here, we illustrate that ACLY is imperative for the invasion and metastasis of colon cancer. Mechanistically, CTNNB1 might be involved in the process.

Overexpression of ACLY has been reported in breast, prostate, bladder, lung, stomach, liver and colon cancers [30–36]. High ACLY expression level is also connected with lymph node metastasis and advanced stages in gastric adenocarcinoma [30]. Phosphorylated ACLY expression levels are a significant factor for predicting a poor prognosis in non–small cell lung cancer, together with clinical stage and tumor size [32]. Silencing ACLY suppresses cancer cell proliferation, induces stemness [37–39], and even promotes tumor cell differentiation and senescence [40–42]. However, there are few reports about the specific role of ACLY in the process of tumor metastasis. MicroRNA-22 abates tumor cell proliferation and invasion and promotes cell apoptosis by inhibiting ACLY [43]. Low molecular weight cyclin E upregulates ACLY enzymatic activity, subsequently promoting transformation, migration and invasion of breast cancer cells [44].

Here, we observe that higher expression level of ACLY in colon cancer cells corresponds with superior migration capability. Deficiency of ACLY results in decreasing total triglycerides and cholesterol synthesis, and reducing the formation of lipid droplet. Meanwhile, we inspect that compared with the control group, the area of metastatic lesion in the lungs and livers shows a significant reduction in the ACLY-knocked-out group. Furthermore, deficiency of ACLY results in changes of epithelial and mesenchymal markers in colon cancer cell lines and nude mouse tissue samples, as EMT is a crucial step in the invasion metastasis process [45].

Previous data demonstrates that AKT upregulates ACLY activity through phosphorylation serine 454 [46]. Interestingly, Hanai J et al. show that inhibition of ACLY “reverses signals” and attenuates phosphatidylinositol 3-kinase (PI3K)/AKT signaling. ACLY knockdown cells show diminished phosphorylation of AKT [38]. Additionally, the crosstalk between the WNT/CTNNB1 and PI3K/AKT signaling has been reported in the development and progression of tumor [47, 48]. Protein kinase glycogen synthase kinase 3β (GSK3β) is not only a downstream of the PI3K/AKT pathway [49], and GSK3β is also a part of the multicomponent complex in the WNT/CTNNB1 pathway, which phosphorylates and subsequently promotes the ubiquitination and degradation of CTNNB1 [50]. Moreover, the nuclear accumulation of CTNNB1, a key member of WNT/CTNNB1 pathway, might indicate higher rate of metastasis in colon cancer [51]. We wonder if ACLY affects the WNT/CTNNB1 signaling pathway, especially CTNNB1, in colon cancer metastasis. Therefore, we detect the protein levels of ACLY and CTNNB1 in colon cancer pathological tissue. Results show that ACLY is positively relative to CTNNB1. This phenomenon is more pronounced in the metastasis subgroup. And patients with high levels of both ACLY and CTNNB1 have poorer prognosis. Coincidently, we find that ACLY combined with CTNNB1. The complex may increase the stability of CTNNB1 protein and facilitate the accumulation of CTNNB1 in the nucleus, subsequently promoting the CTNNB1 transcriptional activity and the migration and invasion abilities of colon cancer.

However, this study has not yet clarified whether the ACLY-CTNNB1 complex could enter cellular nucleus and affect the formation of complexes in WNT/CTNNB1 pathway. Besides, a report has shown ACLY facilitates histone acetylation near double-strand breaks and promotes BRCA1 recruitment and homologous recombination when DNA damage [52]. It is valuable to detect whether ACLY promotes the acetylation of CTNNB1 and the relationship of its phosphorylation and acetylation.

## Conclusion

In this study, we observed that ACLY contributed to colon cancer metastasis in vitro and in vivo. Furthermore, we found that ACLY promoted migration and invasion of colon cancer cells. As ACLY could stabilize and interact with CTNNB1, and assisted CTNNB1 translocation through cytoplasm to nucleus, ACLY may promote the CTNNB1 transcriptional activity and the migration and invasion abilities of colon cancer cells.

## Additional files

Additional file 1: **Table S1.** 'The sequences of target genes. (DOC 55 kb)

Additional file 2: The DNA sequence result of HCT116 KO cells. (DOCX 53 kb)

Additional file 3: The impacts of ACLY deficiency on CTNNB1. (ZIP 4296 kb)

## Data Availability

All data generated or analyzed during this study are included in this published article.

## Abbreviations

ACLY: ATP-citrate lyase
CHX: cycloheximide
Colon cancer: colorectal cancer
CRISPR Cas9: clustered regularly interspaced short palindromic repeats Cas9 system technology
EMT: epithelial-mesenchymal transition
FASN: fatty acid synthase
HE: hematoxylin-eosin staining
HMGCR: 3-hydroxy-3-methylglutaryl-CoA reductase
QPCR: quantitative real-time PCR
SCD1: stearoyl-CoA desaturase 1
SUV: maximum standard uptake value
