# Supplementary material for: ACLY facilitates colon cancer cell metastasis by CTNNB1
Source: J Exp Clin Cancer Res. 2019 Sep 12;38:401. doi: 10.1186/s13046-019-1391-9 (PMC6740040; doi:10.1186/s13046-019-1391-9)
Supplement: Supplementary file 1 — Table S1. 'The sequences of target genes. (DOC 55 kb) [file 13046_2019_1391_MOESM1_ESM.doc]

**Table S1** The sequences of target genes

| Genes | | Primers |
| --- | --- | --- |
| Cyclophilin B (CB) | | F: 5'-AGATGTAGGCCGGGTGATCT-3' |
|  |  | R: 5'-CCGCCCTGGATCATGAAGTC-3' |
| ACLY | | F: 5'-GACTTCGGCAGAGGTAGAGC-3' |
|  |  | R: 5'-TCAGGAGTGACCCGAGCATA-3' |
| CTNNB1 | | F: 5'-GCGCCATTTTAAGCCTCTCG-3' |
|  |  | R: 5'-AAATACCCTCAGGGGAACAGG-3' |
| MMP2 | | F: 5'-GGACTTAGACCGCTTGGCTT-3' |
|  |  | R: 5'-GTGTTCAGGTATTGCATGTGCT-3' |
| E-Cadherin | | F: 5'-GCTGGACCGAGAGAGTTTCC-3' |
|  |  | R: 5'-CAAAATCCAAGCCCGTGGTG-3' |
| N-Cadherin | | F: 5'-GGCGTTATGTGTGTATCTTCACT-3' |
|  |  | R: 5'-CTGACTCCTTCACTGACTCCT-3' |
| Vimentin | | F: 5'-CGGGAGAAATTGCAGGAGGA-3' |
|  |  | R: 5'-AAGGTCAAGACGTGCCAGAG-3' |
| Snail | | F: 5'-TCGGAAGCCTAACTACAGCGA-3' |
|  |  | R: 5'-AGATGAGCATTGGCAGCGAG-3' |
| ZEB1 | | F: 5'-GCCAATAAGCAAACGATTCTG-3' |
|  |  | R: 5'-TTTGGCTGGATCACTTTCAAG-3' |
| Twist1 | | F: 5'-TCCGCGTCCCACTAGCA-3' |
|  |  | R: 5'-AGTTATCCAGCTCCAGAGTCTCT-3' |
| TIMP1 | | F: 5'-GGAATGCACAGTGTTTCCCTG-3' |
|  |  | R: 5'-GGAAGCCCTTTTCAGAGCCT-3' |
| TIMP3 |  | F: 5'-CAGTACCTGCTGACAGGTCG-3' |
|  |  | R: 5'-AGGCGTAGTGTTTGGACTGG-3' |
| TGFβ1 | | F: 5'-TGTCGGGAAATACCACCAGC-3' |
|  |  | R: 5'-GCTCGTCATCGCTTGTTTCA-3' |

| Genes |  | Primers |
| --- | --- | --- |
| TCF4 |  | F: 5'-GATGCTCTGGGGAAAGCACT-3'­ |
|  |  | R: 5'-GTGCCTGCTGAGAGAGATGG-3' |
| Survivin |  | F: 5'-GACGACCCCATGCAAAGGAA-3' |
|  |  | R: 5'-CACCTCTGGTGCCACTTTCA-3' |
| PYGO1 |  | F: 5'-GCCTCGTCCTCCTCCCA-3' |
|  |  | R: 5'- CAGTCCACTATCACCACCTCG-3' |
| PYGO2 |  | F: 5'-GTCGTCGCACTTTGTGGTTG-3' |
|  |  | R: 5'-CTCTTCATTTGCAGACCGGC-3' |
| CCND1 |  | F: 5'-GATCAAGTGTGACCCGGACT-3' |
|  |  | R: 5'-CTTGGGGTCCATGTTCTGCT-3' |
| c-MYC |  | F: 5'-AGCGACTCTGAGGAGGAAC-3' |
|  |  | R: 5'-TGTGAGGAGGTTTGCTGTG-3' |
| Slug |  | F: 5'-GAACTGGACACACATACAGTGAT-3' |
|  |  | R: 5'-ACTCACTCGCCCCAAAGATG-3' |
