# Supplementary material for: ACLY facilitates colon cancer cell metastasis by CTNNB1
Source: J Exp Clin Cancer Res. 2019 Sep 12;38:401. doi: 10.1186/s13046-019-1391-9 (PMC6740040; doi:10.1186/s13046-019-1391-9)

**The DNA sequence** of HCT116 KO cells, which was knockout six base-pairs (CGATTC) and led to no expression of ACLY protein.


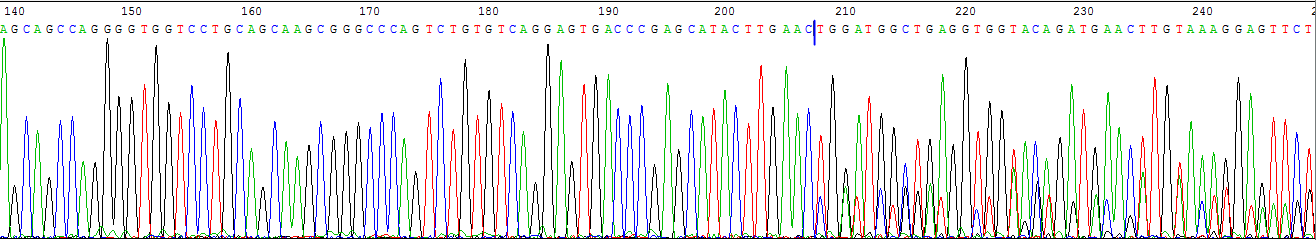


**The DNA sequence result was blasted by NCBI.**


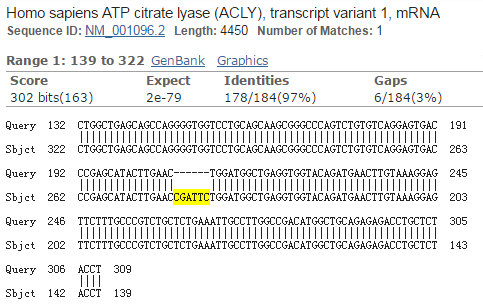

Supplement: Supplementary file 2 — The DNA sequence result of HCT116 KO cells. (DOCX 53 kb) [file 13046_2019_1391_MOESM2_ESM.docx]
