# Supplementary figures and images for: ACLY facilitates colon cancer cell metastasis by CTNNB1
Source: J Exp Clin Cancer Res. 2019 Sep 12;38:401. doi: 10.1186/s13046-019-1391-9 (PMC6740040; doi:10.1186/s13046-019-1391-9)

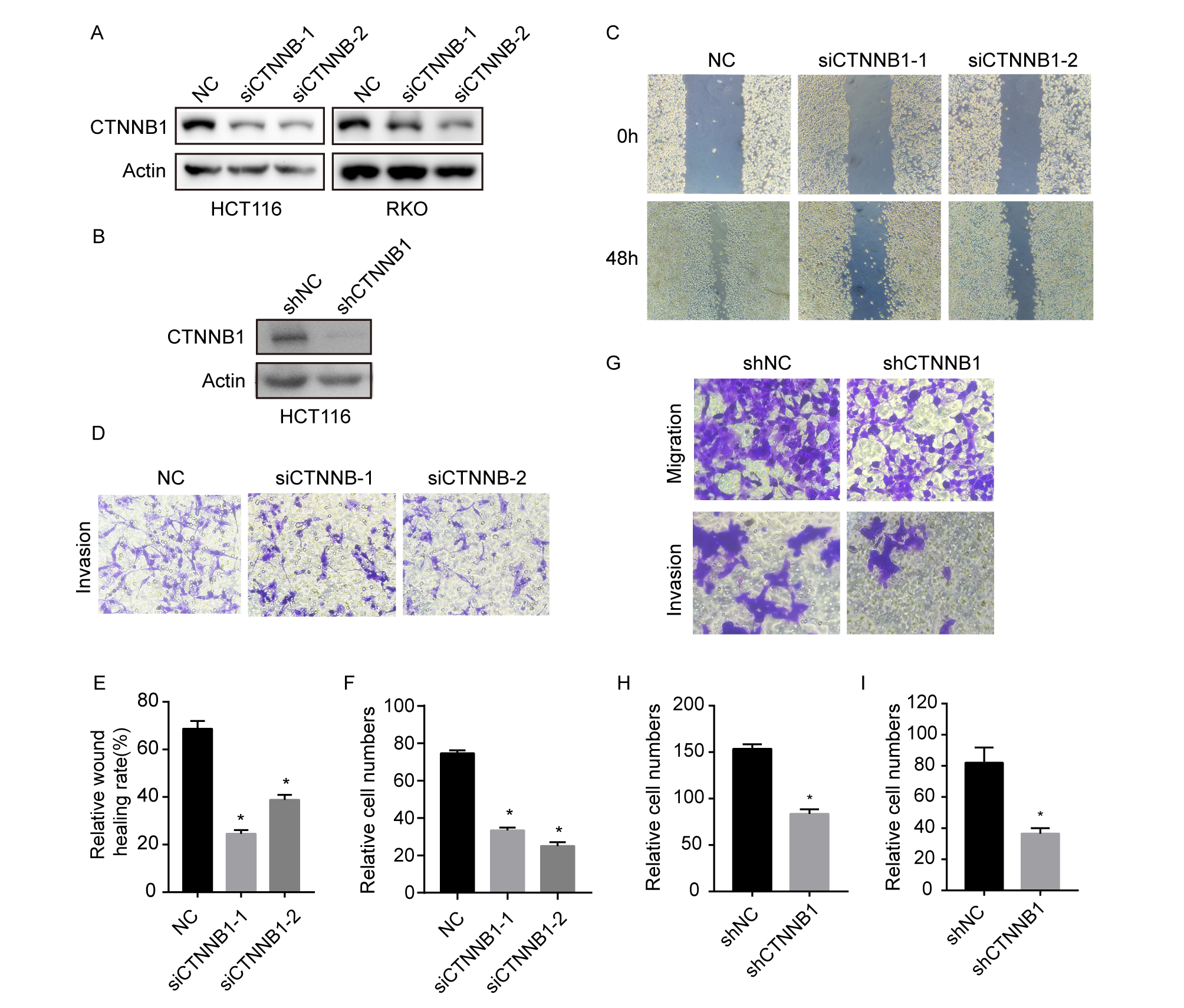

Supplement: Supplementary file 3 — The impacts of ACLY deficiency on CTNNB1. (ZIP 4296 kb) [file 13046_2019_1391_MOESM3_ESM.zip › sFig8(revised)-01.tif]

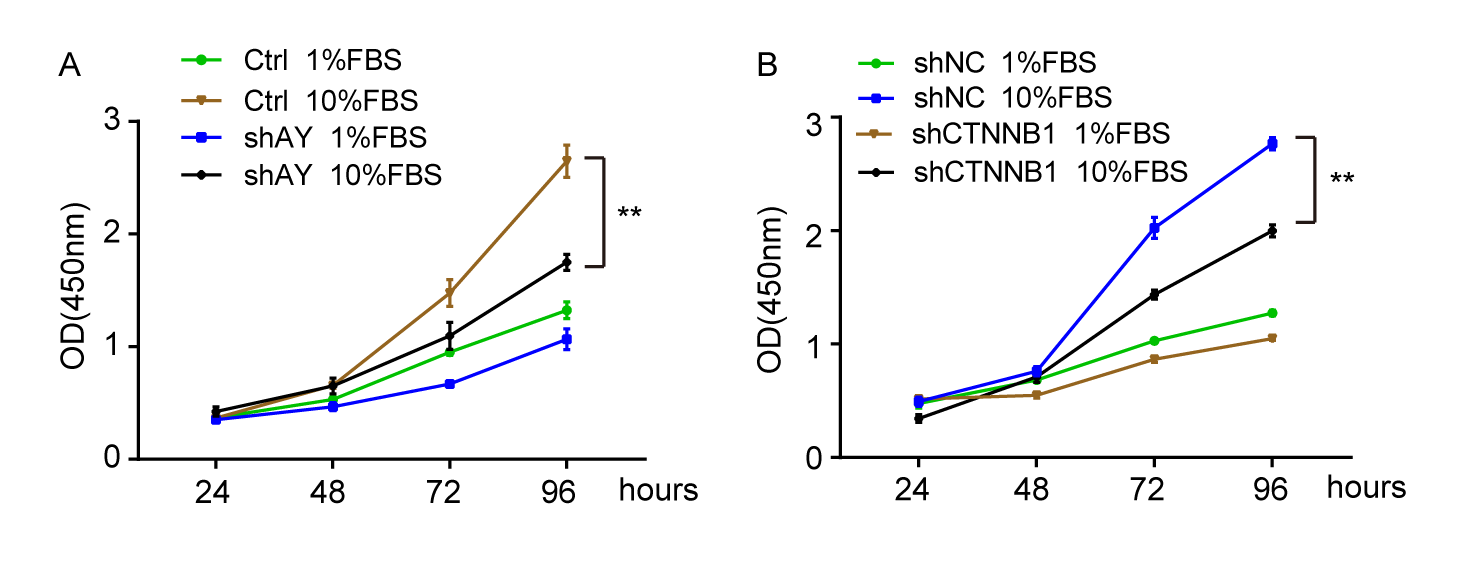

Supplement: Supplementary file 3 — The impacts of ACLY deficiency on CTNNB1. (ZIP 4296 kb) [file 13046_2019_1391_MOESM3_ESM.zip › sFig9 (revised)-01.tif]

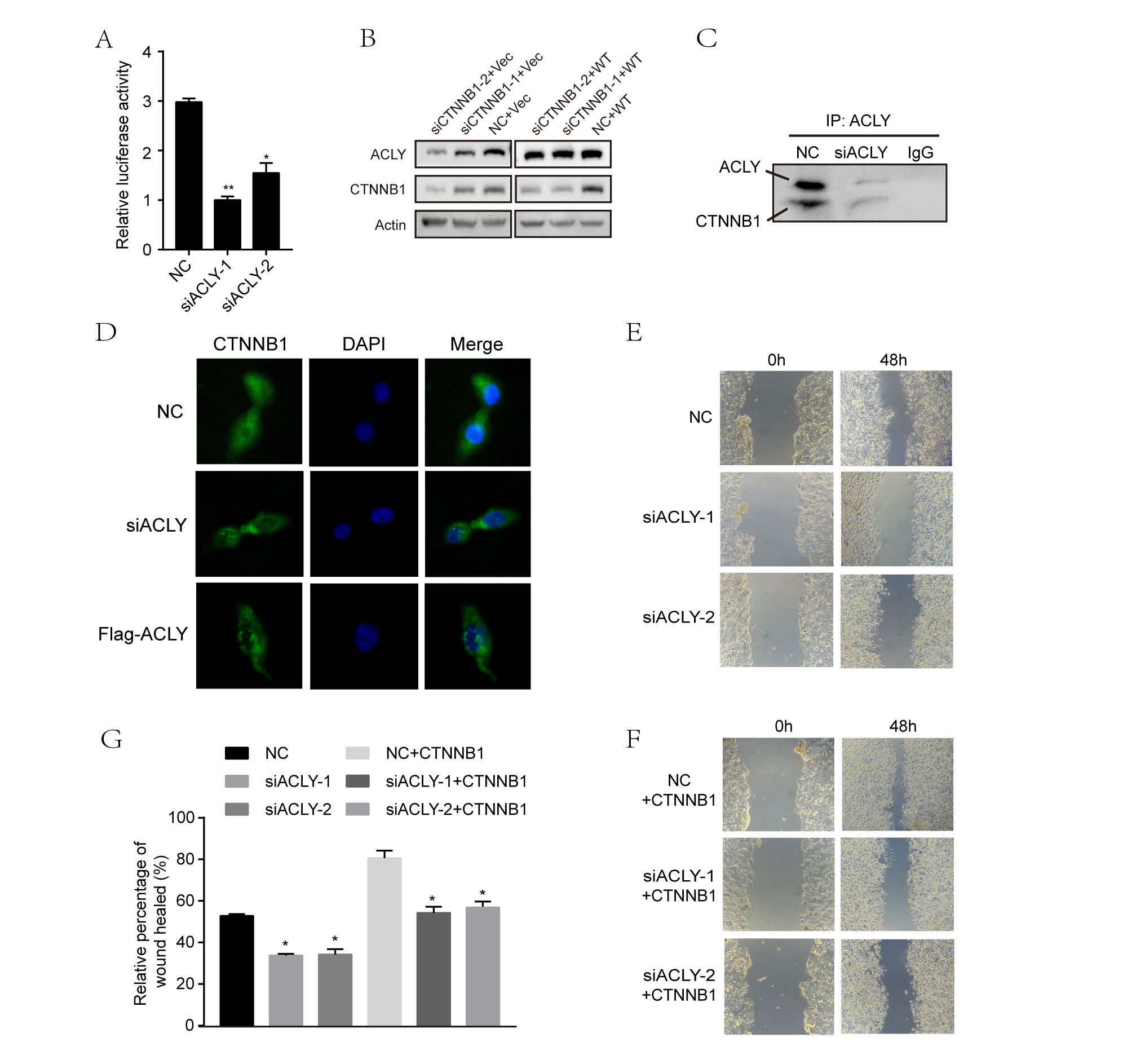

Supplement: Supplementary file 3 — The impacts of ACLY deficiency on CTNNB1. (ZIP 4296 kb) [file 13046_2019_1391_MOESM3_ESM.zip › sFig6 (revised).tif]
