# Supplementary material for: ACLY facilitates colon cancer cell metastasis by CTNNB1
Source: J Exp Clin Cancer Res. 2019 Sep 12;38:401. doi: 10.1186/s13046-019-1391-9 (PMC6740040; doi:10.1186/s13046-019-1391-9)
Supplement: Supplementary file 3 — The impacts of ACLY deficiency on CTNNB1. (ZIP 4296 kb) [file 13046_2019_1391_MOESM3_ESM.zip › Additional File 3 (revised).doc]

**sFig6**

We knocked down ACLY to observe the impacts of ACLY deficiency on CTNNB1 and the changes of cell migration and invasion. Firstly, we performed endogenic immunoprecipitation assay, the results showed that the interaction of ACLY and CTNNB1 weakened after ACLY knocked down (sFig6C). Changes of CTNNB1 cellular location in immunofluorescence staining assay (sFig6D) were consistent with the results of western bolt (Fig6I). Moreover, we found that knockdown of ACLY weakened the abilities of colon cancer cell migration, and overexpression of CTNNB1 could improve in some extent (sFig6E-G).

**(6C)** HEK293T extracts were immunoprecipitated with anti-ACLY or rabbit IgG and immunoblotted by anti-CTNNB1.

**(6D)** After 48 hours of transfection, the location of endogenic CTNNB1 (green) and nuclei (blue, stained by DAPI) in HEK293T cells were analyzed using a fluorescence microscope (magnification, 400×).

**(6E-6G)** HCT116 cells were cotransfected with HA-CTNNB1 or empty vector (as control) plus siRNA-NC or siACLY-1 or siACLY-2 for 48 hours. Wound healing assay (6E and 6F) was performed in HCT116 cells cotransfected with HA-CTNNB1 (or vector as control) and siACLY (or siNC as control). The quantitative analysis of cells wound healing rates to access the migration abilities (6H).

**sFig8**

In order to observe impact of loss-of-CTNNB1 on cancer metastasis, we selected a siRNA sequence of CTNNB1 and constructed HCT116 cells stably knocked down CTNNB1 (shCTNNB1) and the control (shNC). The protein level of CTNNB1 was detected by western blot (Fig2A and 2B). At the same time, we detected the effects of knocking down CTNNB1 on the metastasis ability of RKO cells and HCT116 cells, which was detected by wound healing assay or transwell migration or invasion assay (Fig2C-I). The abilities of migration and invasion weakened in RKO cells transiently knocked down CTNNB1 (Fig2C-2F). Similar changes were observed in HCT116 cells stably knocked down CTNNB1 (Fig2G-2I). These results indicate that loss-of-CTNNB1 could weaken the colon cancer cell migration and invasion abilities.

**(8A)** Western blot analysis to access the knockdown efficiency of CTNNB1 siRNA (siCTNNB1-1 and siCTNNB1-2) in HCT116 cells and RKO cells, and siRNA-NC as the control.

**(8B)** Western blot analysis to access the knockdown efficiency of shCTNNB1 in HCT116 cells, and shNC as the control.

**(8C)** Wound healing assays to access the effects of CTNNB1 deficiency on the migration ability of RKO cells.

**(8D)** Transwell invasion assays to evaluate the effects of CTNNB1 deficiency on the invasion ability of RKO cells.

**(8E and 8F)** The quantitative analysis of wound healing rates (8E). The quantitative analysis of cells across the transwell membrane (8F).

**(8G-8I)** Transwell migration and invasion assays to detect the effects of CTNNB1 stably knockdown on the metastasis ability of RKO cells (8G). The quantitative analysis of cells across the transwell membrane (8H and 8I).

**sFig9**

Since tumor cells were cultured with 1% serum in migration and invasion assays, the growth rates of tumor cells weakened drastically. Moreover, we performed cell counting kit-8 (CCK8) assay to detected tumor cell growth rates. As shown in sFig9A and 9B, there was significant difference of tumor cell growth rates in the 10% serum cultured condition between the control group and gene deficiency group. Whereas, the difference weakened drastically when cells were cultured in 1% serum. Cell cultured condition with 1% FBS is similar with the microenvironment of tumor cells in vivo, where there is short of nutrients. Taking together, there is little effects of cell growth rates on the invasion, migration, and in vivo tumor formation ability of the shRNA knockdown cells.

(9A and 9B) HCT116 cells that ACLY was stably knockout and HCT116 cells that CTNNB1 was stably knockdown were seeded in 96-well plates and cultured with 1% or 10% serum for 24, 48, 72, 96 hours. Cell viability was measured using cell counting kit-8 (BIMAKE, B34304). The optical density was measured at 450 nm.
